# Lifespan cardiometabolic exposures are associated with midlife white matter microstructure: The Bogalusa Heart Study

**DOI:** 10.1002/alz.089817

**Published:** 2025-01-09

**Authors:** Reagan T Dugan, Sreekrishna Ramakrishnapillai, Julia St Amant, Kori Murray, Kevin McKlveen, Maryam Naseri, Kaitlyn Madden, Lydia Bazzano, Owen T. Carmichael

**Affiliations:** ^1^ Pennington Biomedical Research Center, Baton Rouge, LA USA; ^2^ Louisiana State University, Baton Rouge, LA USA; ^3^ Tulane University School of Public Health and Tropical Medicine, New Orleans, LA USA

## Abstract

**Background:**

Compartment model analysis of diffusion MRI data provides unique information on the microstructural properties of white matter in the brain. However, studies relating compartment model microstructural measures to longitudinal cardiometabolic health data are rare.

**Method:**

In this study, 130 cognitively healthy participants in the Bogalusa Heart Study (age 55.4 ± 4.5) completed high angular resolution diffusion MRI scans. Neurite orientation dispersion and density imaging (NODDI) and diffusion tensor analysis were performed, and values of neurite density, orientation dispersion, free water fraction, fractional anisotropy, and mean diffusivity were measured in ROIs of highly organized and diffuse white matter. Multiple linear regression models were used to relate the white matter microstructure metrics to demographics and cardiometabolic risk factors.

**Result:**

Organized tracts showed significantly greater NDI, and FWF, and significantly lower ODI, than the diffuse white matter. In organized white matter greater age was associated with greater FWF and lesser NDI, women had lower FWF than men, and African Americans had greater ODI than whites. Worse cardiometabolic measures and brain health were generally associated with lesser NDI. Greater blood pressure in pre‐adulthood was associated with lesser NDI. Within the diffuse white matter, greater age was associated with greater FWF, women had lesser FWF than men, and African Americans had lesser NDI and greater ODI than whites. Worse cardiometabolic measures and brain health were generally associated with greater FWF.

**Conclusion:**

In a racially diverse, cognitively healthy, middle‐aged community cohort sample, summary measures from diffusion tensor and compartment model analyses of multi‐shell diffusion MRI data were associated cardiometabolic risk factors from youth to midlife as well as demographic factors.

